# Live-cell omics with Raman spectroscopy

**DOI:** 10.1093/jmicro/dfaf020

**Published:** 2025-04-24

**Authors:** Ken-ichiro F Kamei, Yuichi Wakamoto

**Affiliations:** Department of Basic Science, Graduate School of Arts and Sciences, The University of Tokyo, 3-8-1 Komaba, Meguro-ku, Tokyo 153-8902, Japan; Department of Basic Science, Graduate School of Arts and Sciences, The University of Tokyo, 3-8-1 Komaba, Meguro-ku, Tokyo 153-8902, Japan; Research Center for Complex Systems Biology, The University of Tokyo, 3-8-1 Komaba, Meguro-ku, Tokyo 153-8902, Japan; Universal Biology Institute, The University of Tokyo, 3-8-1 Komaba, Meguro-ku, Tokyo 153-8902, Japan; Collaborative Research Institute for Innovative Microbiology, The University of Tokyo, 1-1-1 Yayoi, Bunkyo-ku, Tokyo 113-8657, Japan

**Keywords:** live-cell omics, Raman spectroscopy, single-cell analysis, cellular plasticity

## Abstract

Genome-wide profiling of gene expression levels in cells, such as transcriptomics and proteomics, is a powerful experimental approach in modern biology, allowing not only efficient exploration of the genetic elements responsible for biological phenomena of interest, but also characterization of the global constraints behind plastic phenotypic changes of cells that accompany large-scale remodeling of omics profiles. To understand how individual cells change their molecular profiles to achieve specific phenotypic changes in phenomena such as differentiation, cancer metastasis and adaptation, it is crucial to characterize the dynamics of cellular phenotypes and omics profiles simultaneously at the single-cell level. Especially in the last decade, significant technical progress has been made in the *in situ* identification of omics profiles of cells on the microscope. However, most approaches still remain destructive and cannot unravel the post-measurement dynamics. In recent years, Raman spectroscopy-based methods for omics inference have emerged, allowing the characterization of genome-wide molecular profile dynamics in living cells. In this review, we give a brief overview of the recent development of imaging-based omics profiling methods. We then present the approach to infer omics profiles from single-cell Raman spectra. Since Raman spectra can be obtained from living cells in a non-destructive and non-staining manner, this method may open the door to live-cell omics.

## Teaser

This review highlights recent advances in Raman spectroscopy-based omics inference methods and clarifies their advantage over existing omics profiling methods for achieving non-destructive and genome-wide molecular profiling of live cells at the single-cell level. It also discusses the potential of Raman-based omics profiling to reveal the omics constraints that are critical for understanding cellular plasticity.

## Introduction

Cells have the ability to change and adapt their phenotype in response to their environment and context. This ability is known as ‘cellular plasticity’ or ‘phenotypic plasticity’ and is responsible for processes such as the development of multicellular systems, cancer metastasis, and physiological adaptation and survival to stressful conditions [1–7]. Plastic changes in cellular phenotypes are often accompanied by large-scale remodeling of gene expression and metabolic profiles. Therefore, understanding the molecular events that drive phenotypic changes and the system-level constraints behind the global remodeling requires unraveling the dynamics of numerous biomolecular species within cells.

Fluorescent proteins are by far the most widely used reporters for measuring the dynamics of biomolecular abundances inside living cells [8–10]. The availability of fluorescent proteins with diverse excitation and emission spectra has enabled the simultaneous abundance quantification of multiple protein species in living cells [11–17]. Quantification is not limited to proteins; ratiometric measurements such as Förster resonance energy transfer can reveal the dynamics of small biomolecules inside cells, including metabolites [18–23]. However, despite recent significant advances in multiplexed imaging [24–32], the number of biomolecular species that can be tracked simultaneously is still limited in most practical settings. Unraveling the dynamics of comprehensive biomolecular profiles inside living cells therefore remains a major technical challenge in the life sciences.

Omics technologies such as transcriptomics, proteomics and metabolomics have been used to characterize comprehensive molecular profiles of cells. In addition to standard methods using DNA sequencers and mass spectrometers, there is a growing trend towards multiplexed identification of RNA and protein species *in situ* in fixed cell samples directly on the microscope [26,27,31,33–37]. Despite their widespread and significant contributions and applications to biological studies, these methods are inherently destructive. Therefore, the characterization of the dynamics of numerous biomolecules in living cells requires new measurement principles.

In this review, we first give a brief overview of the recent development of transcriptome measurements that identify thousands of target transcripts in single cells directly on the microscope. Second, we present approaches to non-destructive omics measurements that extract a small fraction of the cytosolic contents of living cells without killing them. Third, we review Raman spectroscopic approaches to reveal multi-omics profiles and how they can be exploited to achieve live-cell omics measurements. Finally, we discuss the advantages and current limitations of Raman spectroscopic omics profiling.

## Imaging-based *in situ* single-cell transcriptomics

Comprehensive gene expression profiles in single cells are evaluated by quantifying the abundance of genome-wide protein species (proteomics) or RNA species (transcriptomics). Standard proteomics and transcriptomics protocols require extraction and purification of target molecules (proteins/RNAs) from cell or tissue samples for subsequent analysis by mass spectrometry or next-generation sequencing. However, especially in the last decade, techniques have been established to quantify the abundance of different RNA species *in situ* directly on a microscope without extracting these molecules from samples [33–43].

The development of imaging-based transcriptomics techniques has been driven by a surge of interest in spatial omics, an omics technology that unravels the omics profiles of individual cells or small tissue regions by preserving their spatial information [33–37]. The analysis provides an understanding of the spatial organization of cell populations, which is critical to the function of many biological systems, such as organs.

**Fig. 1.**
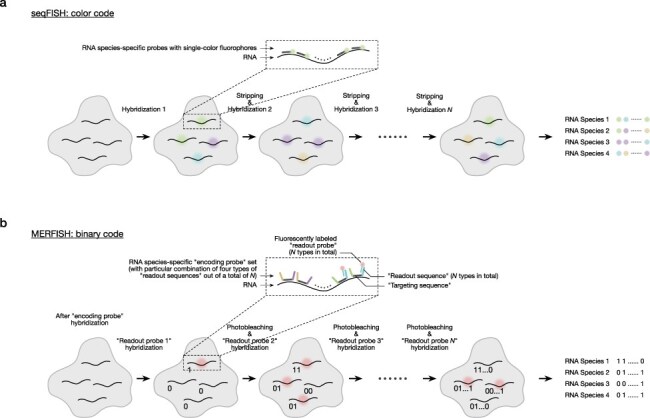
Two representatives of highly multiplexed smFISH methods.

For imaging-based transcriptomics, the critical technical element is single-molecule fluorescent *in situ* hybridization (smFISH), which allows the detection of fluorescently probed transcript RNA with single-molecule resolution within fixed cells [44–46]. Lubeck et al. introduced an approach of smFISH called sequential FISH (seqFISH), which significantly increases the number of detectable RNA species by barcoding transcripts with multiple colors and cycling probe hybridization and stripping (Fig. 1a)[38,39].

(a) seqFISH. RNA species-specific probes are designed and color-coded with fluorophores. Repeated hybridization and stripping of the probes results in discernible RNA species-specific color combinations, allowing the identification of transcripts at their original location. (b) MERFISH. RNA species-specific encoding probe sets are designed to have a particular combination of readout sequences to allow binary coding of each type of transcript. By hybridizing fluorescently labeled readout probes to readout sequences in the encoding probes and photobleaching repeatedly, each transcript can be identified in its original location.

Chen et al. developed another smFISH-based method called multiplexed error-robust FISH (MERFISH) [40]. Instead of color barcoding, MERFISH assigns a binary code to each type of transcript using unique encoding probes (Fig. 1b). In addition to sequences that bind the target RNA, the probes contain readout sequences that bind fluorescently labeled readout probes. Multiple rounds of readout probe hybridization and photobleaching can read the binary codes of many transcripts and identify them within cells. Furthermore, by using a subset of the $2^N -1$ binary codes of length *N* (*N* rounds) separated by a certain Hamming distance, the identification can be made robust against readout errors. An advantage of MERFISH is that hybridized probes do not need to be stripped in each round to quench fluorescence signals. As a result, sequential hybridization rounds can be performed quickly.

Many other barcoding and imaging-based transcriptomics techniques have been actively developed to overcome technical challenges such as low signal-to-noise ratio and spatial resolution [41–43]. This review does not cover the latest topics in imaging-based transcriptomics; readers should refer to more specific reviews, such as [33–36].

## Transcriptomics with non-destructive extraction of cellular contents

smFISH-based transcriptomics requires the fixation of cell and tissue samples for probe hybridization. Therefore, the method is inherently destructive and cannot unravel how cells subsequently change their states from identified transcriptomic states.

To overcome this inherent invasive nature of the smFISH-based method, as well as standard next-generation sequencing-based methods such as single-cell RNA sequencing (scRNA-seq), several statistical methods have been proposed to elucidate the transcriptome dynamics from the snapshot measurements [47–50]. For example, RNA velocity quantifies the abundance of unspliced and spliced mRNAs in scRNA-seq data and estimates the directions in which high-dimensional gene expression states evolve [51,52]. Another example, called Waddington-OT, reconstructs cell state trajectories from multiple time-point scRNA-seq data [53]. Using the mathematical framework of optimal transport, Waddington-OT can infer probabilistic trajectories of gene expression profiles even for non-stationary processes such as reprogramming of fibroblasts to induced pluripotent stem cells (iPSCs).

These snapshot-based approaches are indeed powerful and useful. They have successfully identified key transcriptome factors that contribute to fate decision and reprogramming [51–53]. However, some of the assumptions behind the methods are not necessarily validated [50,52]. Therefore, whether inferred transcriptomic dynamics represent real changes in individual cells needs to be scrutinized in each system.

**Fig. 2.**
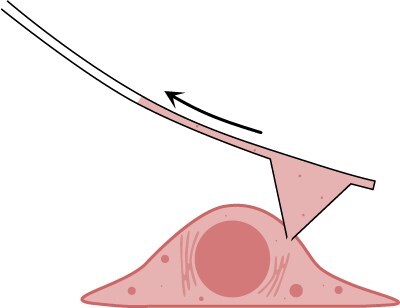
Live-seq.

To go beyond statistical inference of omics profile dynamics, Chen et al. have developed a method called Live-seq, an approach that extracts RNA molecules from single cells while preserving their viability [54]. The technique uses a cantilever-like probe in which microscopic fluid channels are fabricated [55,56]. The probe has a small, sub-micron aperture that enables cytoplasmic biopsies at the single-cell level (Fig. 2). Interestingly, extraction of  1 pL of cytoplasm (about 1/10 of the total cell volume of typical mammalian cells such as fibroblasts) does not significantly affect the viability of several types of mammalian cells; 85–89% of the cells remain viable. The extracted cytoplasm is further processed using a sensitive RNA-seq method such as Smart-seq2 [57]. Even from such a small volume of extracts, Live-seq can detect an average of 2100 genes, which is sufficient to classify several different cell types and states.

In this method proposed by Chen et al. [54], transcripts are collected from living single cells by picoliter-scale cytoplasmic biopsy using a cantilever-like probe. The transcripts in the collected cytoplasm are analyzed by a sensitive RNA-seq method such as Smart-seq2 [57].

The main advantage of Live-seq is that it allows multiple time point biopsies from the same cells. Therefore, one can directly link the profiles at different time points to unravel the transcriptomic history of individual cells.

Despite its promising capability to characterize transcriptome dynamics at the single-cell level, Live-seq still has several limitations. For example, due to the small volume of the extract, the rate of passing the quality control defined by several criteria, such as the number of detected genes, was not very high (<50%). Another potential limitation is the disturbance caused by the cytoplasmic biopsy. Although most cells remain viable and transcriptomic profiles are largely unaffected, cytoplasmic biopsy leads to a temporal reduction of cell volume by 40–70%. Therefore, the absence of confounding effects should be carefully evaluated in any targeted cell system. These issues would be partially resolved with more sensitive scRNA-seq methods such as Quartz-Seq2 [58,59], since they contribute to the detection of more genes and allow minimizing the cytoplasmic extraction volume. However, it remains to be clarified whether analysis with a smaller volume of cytoplasmic content collected from spatially heterogeneous cytoplasm preserves the trend of a comprehensive transcriptomic profile.

## Raman spectroscopy for cellular phenotyping

The omics analysis methods described above rely on direct visualization or quantification of target molecules. On the other hand, recent years have seen the emergence of methods that use cell imaging or spectroscopy to obtain information that is highly correlated with omics profiles and to infer omics profiles based on this information [60–64]. In this article, we highlight the technique of omics inference using Raman spectroscopy.

**Fig. 3.**
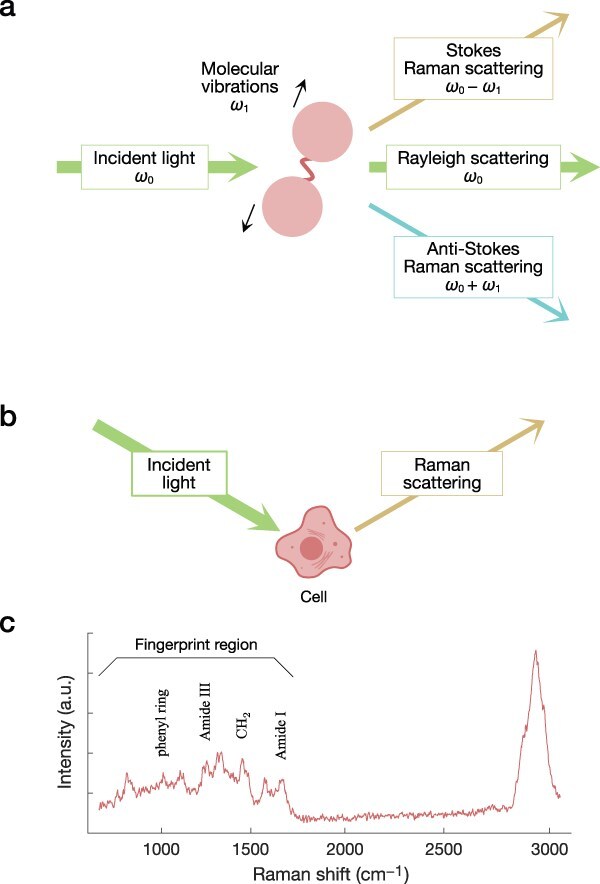
Raman scattering.

Raman scattering is the inelastic photon scattering phenomenon resulting from the interaction of incident light with target molecules [65–76]. When the target molecules are exposed to monochromatic light, most of the scattered photons have the same energy (wavelength) as the incident light, which is called Rayleigh scattering. On the other hand, a small fraction (~1 in 10^6^–10^8^) of the scattered photons gain or lose energy depending on the vibrational modes of the chemical bonds in the target molecules (Fig. 3a) [66,67,69,72]. This phenomenon was first discovered by Raman and Krishnan in 1928 [77,78] and is called Raman scattering.

(a) Rayleigh and Raman scattering. Incident light on a molecule is scattered and the scattering can be classified into two categories: Rayleigh scattering and Raman scattering. While Rayleigh-scattered light has the same energy as the incident light, Raman-scattered light is composed of photons with different energy levels depending on the targeted molecular vibrations. (b) Raman spectroscopy of biological cells. By exposing a biological cell to incident light, Raman-scattered light from the cell is obtained. If the incident light is monochromatic, analyzing the Raman spectrum can provide information about the molecular composition of the target cell. (c) An example of a cellular Raman spectrum. This Raman spectrum is obtained from a *S. aureus* cell. The spectral region from 700 to 1800 cm^−1^, where the signals of most biomolecules such as proteins and metabolites are observed, is called the fingerprint region.

The phenomenon is called Stokes Raman scattering when the energy of the scattered photons is lower than that of the incident photons, and anti-Stokes Raman scattering when the energy of the scattered photons is higher (Fig. 3a). Since the energy shift depends on the chemical bonds in the target molecules, a Raman spectrum can be viewed as a fingerprint of the target molecules and used to identify them.

By combining Raman spectroscopy with a microscope, Raman spectra can be obtained from microscopic samples such as biological cells [65]. This method is called micro-Raman spectroscopy or Raman microscopy. Raman microscopy is a non-labeling and non-destructive method. Therefore, Raman spectra can be obtained even from single living cells (Fig. 3b). Prominent peaks in Raman spectra of cells correspond to signals from chemical bonds that are abundant in cells, such as C-H, C-C, and CH_2_ (Fig. 3c) [66,72,75].

Some biomolecules have prominent Raman spectral peaks that are distinguishable from the other peaks. In such cases, the abundance of specific biomolecules in cells can be estimated from the signal intensity of the peaks [76]. For example, peak quantification has been used to estimate the amount of biomolecules such as cytochrome *c* and lipids in individual cells based on their characteristic spectral peaks at the Raman shift of 750 and 2850 cm^−1^, respectively [68,79].

Even when the correspondence between Raman spectral peaks and molecules is not obvious, changes in specific peaks in cellular Raman spectra can sometimes be linked to the physiological signatures of target cells. For example, Huang et al. monitored Raman spectral changes in the fission yeast *Schizosaccharomyces pombe* induced by the respiratory inhibitor KCN and showed that the Raman spectral peak at 1602 cm^−1^ disappears along with physiological changes leading to death [80]. Since this peak was also weak in cells under nutrient-depleted conditions, this Raman spectral peak was proposed as a signature of life in *S. pombe*.

Germond et al. measured Raman spectra of antibiotic-resistant *Escherichia coli* strains obtained by experimental evolution under exposure to different types of antibiotics [81]. They identified Raman spectral peaks that correlated with antibiotic resistance against different modes of action and with the expression levels of some genes that contribute to antibiotic resistance.

In contrast to these spectral peak-based interrogations of cellular states, an alternative approach has also been used that analyzes the entire Raman spectrum of a cell as high-dimensional data and links it to cell types and phenotypes through appropriate dimensional reduction and classification [64,66,71,73,74,76,82–87]. In principle, cellular Raman spectra reflect the whole molecular composition of cells. Therefore, they potentially contain rich information that allows us to distinguish essential differences of target cells. In fact, this approach has been successfully applied to elucidate cell state transitions during differentiation [85], rapid identification of pathogenic bacteria [87] and tumor diagnosis [71,73,84]. For example, Ichimura et al. showed that principal component analysis of cellular Raman spectra obtained from cells undergoing differentiation from mouse embryonic stem cells can distinguish between undifferentiated and differentiated states [85].

When thousands of Raman spectral data are available for analysis, more sophisticated machine learning methods can be used to learn the correspondence between Raman spectra and cell types. In fact, Ho et al. obtained 60 000 spectra from 30 pathogenic bacterial and yeast species and trained a convolutional neural network (CNN) to classify them [87]. Even with a low signal-to-noise ratio of the Raman spectral data used in the analysis, they showed that Raman spectra could identify these 30 species with an average accuracy of 82%. For the task of classifying the antibiotics that should be used to treat the infection, the accuracy was 97%. Furthermore, when the task was a binary classification between methicillin-sensitive *Staphylococcus aureus* and methicillin-resistant *S. aureus* (MRSA), the classification accuracy of the trained CNN was 99%. These results demonstrate that global patterns of cellular Raman spectra from different bacterial species and strains contain unique identification signals.

## Raman-omics correspondences and live-cell omics with Raman spectroscopy

Classification of biological species and phenotypes based on cellular Raman spectra and machine learning has significant application potential for screening and diagnosis. However, it would be essential to know the molecular underpinnings in order to use the resulting classification reliably. It is then natural to ask whether omics profiles can also be predicted from cellular Raman spectra.

Intuitively, such a task seems daunting, because a cell contains an enormous variety of biomolecules. For example, even a single bacterium, *E. coli*, contains  4300 protein-coding genes [88–90], and human cells are predicted to contain  20 000 protein-coding genes [91]. This means that changes in the gene expression profiles of *E. coli* cells and human cells are represented as trajectories in high-dimensional state spaces with 4300 and 20 000 dimensions, respectively.

However, despite such high-dimensional state spaces, many omics and bioinformatics studies have revealed that gene expression levels are correlated genome-wide and that the effective dimensionality of comprehensive gene expression profiles in cells is strongly constrained to low-dimensional manifolds [92–102]. For example, Keren et al. have shown that only a few scaling factors are sufficient to describe genome-wide gene expression profiles across conditions in *Saccharomyces cerevisiae* and *E. coli* [95]. Furthermore, Biswas et al. have shown that whole transcriptome profiles in human cells can be reconstructed from the expression level information of 100 genes [100]. Effective low dimensionality of gene expression profiles has already been actively exploited in omics analyses, such as accurate determination of transcriptome profiles with reduced coverage depth in next-generation sequencing [99] and spatial omics [103]. For example, the method called Tangram first learns the relationship between the expression profiles of selected genes and those of genome-wide genes, and can reconstruct spatial maps of global transcriptome profiles based on spatially resolved expression profiles of the selected genes measured by methods such as MERFISH [103].

With such strong constraints on global gene expression profiles, it may be possible to infer omics profiles of target cells from their Raman spectra. Indeed, evidence for such Raman spectroscopic omics is accumulating.

**Fig. 4.**
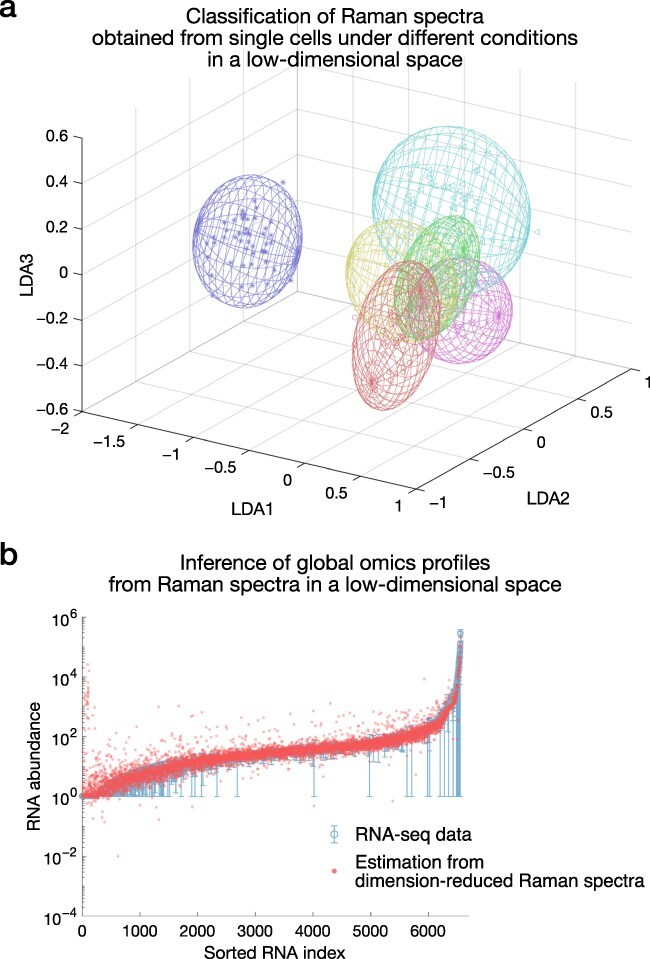
Dimensional reduction of cellular Raman spectra and global transcriptomic inference from Raman spectra (adapted from [60] with modifications).

Kobayashi-Kirschvink et al. cultured fission yeast *S. pombe* cells under 10 different culture conditions and obtained Raman spectra from  60 individual cells per condition [60]. By applying principal component linear discriminant analysis [82,83,86] to the Raman spectra, they reduced the dimensionality of the Raman spectra and showed that the cellular Raman spectra from different conditions form distinct clusters in the dimension-reduced space (Fig. 4a). Therefore, the cellular Raman spectra contain information about the differences in cellular state under 10 culture conditions. Next, they measured the transcriptome profiles of the cell populations under the same conditions and inferred the correspondence between the dimension-reduced Raman spectra and the transcriptome profiles by partial least squares regression [104]. Using the correspondence, they showed that the condition-dependent transcriptomic changes can be estimated from the dimension-reduced Raman spectra (Fig. 4b), whose estimation errors were evaluated by leave-one-out cross-validation and permutation test.

(a) Classification of single-cell Raman spectra in a low-dimensional space. *S. pombe* cells were cultured under different conditions and their single-cell Raman spectra were measured. The spectra were classified into a space with a lower dimensionality than the original spectra. Each point represents a dimensionally reduced single-cell Raman spectrum, and different markers and colors correspond to different culture conditions. (b) An example of the inference of global transcriptomic profiles from Raman spectra. Once the relationship between cellular Raman spectra and transcriptomes has been obtained using training conditions, transcriptomic profiles can be calculated from Raman spectra for other conditions. In [60], condition-dependent global changes in transcriptomes were inferred with reasonable accuracy. Here, the result of the transcriptomic inference of *S. pombe* in the YE condition is shown. Blue points represent the expression levels of the transcripts measured by RNA-seq (average of the two replicate measurements with error bars showing the maximum-minimum range), sorted from low to high along the horizontal axis. Red points represent expression levels predicted from Raman spectra.

The same approach has been applied to infer the transcriptomes of *E. coli* [60] and MRSA strains with different levels of resistance to vancomycin, a frontline antibiotic used to treat MRSA infections [64]. In the case of the transcriptome inference of the MRSA strains in [64], all MRSA strains were cultured under the same conditions. Thus, the transcriptomic differences result from the genotypic differences between the strains. Therefore, cellular Raman spectra can also predict genotype-dependent transcriptomic changes.

Raman spectroscopic omics inference is not limited to transcriptomics. Using quantitative proteomic data from *E. coli* under 15 different culture conditions [105], Kamei et al. showed that condition-dependent changes in proteome profiles can also be inferred from Raman spectra [63]. This result suggests that multi-omics profiles can be obtained simultaneously from cellular Raman spectra if the correspondences between Raman and multi-omics profiles are determined beforehand.

**Fig. 5.**
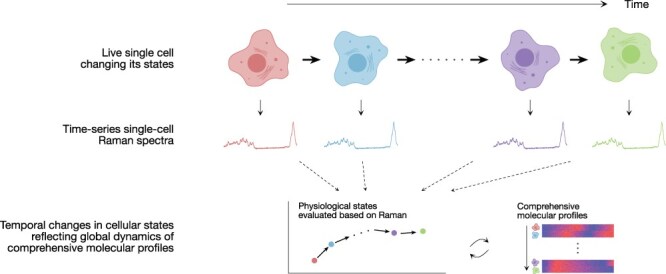
Live-cell omics with Raman spectroscopy.

The above-mentioned correspondences between Raman spectra and omics profiles have been investigated using population-averaged Raman spectra and omics profiles. Recently, Kobayashi-Kirshvink et al. have extended this linkage to the single-cell level and developed a method called Raman2RNA [62]. One of the approaches they reported uses anchor genes representing different cell types that arise during reprogramming of mouse fibroblast cells to iPSCs. By obtaining both Raman spectra of cells and smFISH on the nine anchor genes from thousands of individual cells at multiple time points during reprogramming, they trained a neural network to predict smFISH anchors from the Raman profiles. They then used Tangram [103] to recover full scRNA-seq profiles from the predicted expression levels of the anchor genes. Furthermore, they applied this method to time-lapse measurements during the differentiation of mouse ES cells treated with retinoic acid. After confirming the accuracy of the transcriptome profiles predicted from the Raman spectra, they performed Raman time-lapse measurements for single cell lineages to reveal the dynamics of their transcriptome profiles (Fig. 5). They detected ectoderm versus extraembryonic endoderm (XEN)-like cell differentiation as early as 48 h, which is not readily detectable by scRNA-seq [106]. Thus, this study demonstrated that Raman spectroscopy-based live-cell omic profiling is indeed possible.

Time-lapse Raman measurements of single-cell lineages enable us to directly observe temporal changes in cellular states that are inextricably linked to the global dynamics of comprehensive molecular profiles.

## Limitations and future perspectives

Since Raman spectroscopy is a non-labeling and non-destructive method, the correspondence between cellular Raman spectra and omics profiles paves the way for live-cell multi-omics profiling that can unravel the dynamics of the comprehensive molecular composition of cells along with their phenotypic history. Such information would be critical for understanding the cellular plasticity that underlies phenomena such as stem cell differentiation, cancer metastasis and adaptation.

Why do we measure omics information at all? A widely shared motivation for omics measurement is to efficiently narrow down the omics components that significantly change their abundance in specific cell types or environments. These responsive elements among thousands of omics components may well be the leading candidate factors responsible for determining cell types or generating specific biological phenomena. Another important motivation for measuring omics profiles is to elucidate the global architecture and constraints in omics profiles, such as abundance distributions [107], resource allocation to different intracellular functions [96,105,108,109] and correlation networks between omics components [63]. Crucially, it has been shown that these system-level properties are tightly linked to key physiological properties of cells, such as growth [96,105,108,109] and phenotypic plasticity [110–113], which may not be attributable to a small set of omics elements. Instead, coarse-grained macroscopic properties of omics profiles may have greater explanatory power for these physiological properties. For the first type of omics analysis, the precision of the measurement of each component is critical, while for the second type of omics analysis, the unbiased coarse-grained structure may be more important than the precision of the abundance of each component. Since it exploits the effective low dimensionality of omics profiles, Raman-based omics inference may perform better for the second type of analysis.

Finally, we note several current limitations of Raman-based live-cell omics.

First, this approach does not directly quantify the abundance of transcripts and proteins. Therefore, the statistical correspondence between Raman spectra and omics profiles must be known in advance for Raman-based omics inference. Due to this statistical nature, the resulting inferred omics profiles depend on the datasets used to learn the correspondence.

Second, the low efficiency of Raman scattering (~1 in 10^6^–10^8^ scattered photons [66,67,69,72]) leads to a low signal-to-noise ratio of the spectra, which inevitably affects the resulting inferred omics profiles. Spontaneous Raman measurements typically require relatively long exposures of target cells to laser light (on the order of seconds) to obtain reasonably intense Raman signals. Consequently, photodamage to cells from incident light must be considered, especially when performing time-lapse Raman measurements on living cells. This problem might be partially resolved by employing nonlinear Raman scattering methods such as coherent anti-Stokes Raman scattering and stimulated Raman scattering (SRS) [69,72,114–120]. These methods are based on nonlinear optical processes resulting from the interaction between two lasers. By adjusting the frequency difference between the two lasers, the Raman signals at a given wavenumber are enhanced by several orders of magnitude. As a result, Raman signals of targeted Raman shift can be acquired with significantly shorter exposure time. In these methods, Raman signal enhancement occurs at a specific wavenumber. Consequently, obtaining Raman spectra over a wide range of wavenumbers requires rapid frequency switching. Several methods, such as multicolor SRS [119], have been developed to overcome this problem. Therefore, the application of these nonlinear Raman methods to omics inference would be an important direction to explore.

Third, again because of the weak Raman signals, the method cannot be applied to biological samples that emit strong autofluorescence. This problem can be partially circumvented by using longer wavelength incident light; the use of red to infrared light tends to reduce the autofluorescence of biological samples. However, the intensity of Raman signals also decreases in inverse proportional to the fourth power of the wavelength [68]. Therefore, some biological samples may not allow reliable Raman measurements.

Fourth, a standard optical configuration and measurement procedure have not yet been established. As a result, it remains difficult to compare and combine Raman signals obtained with different microscopes to infer omics profiles. It should also be noted that Raman signals are sensitive to the environmental conditions around the microscopes. Therefore, besides specifying factors that affect the stability and reproducibility of Raman measurements [121], it is crucial to develop efficient calibration methods to correct the signal variations caused by differences and instabilities of the optical setup [121–123].

Fifth, the quality and precision of inferred omics profiles may be affected by the spatial resolution and spectral resolution of Raman measurements. However, no systematic analysis has been performed to determine their effect on omics inference. Some studies collected one Raman spectrum per single cell by adjusting the size of an area from which Raman signals are collected close to the cell size and lowering the spatial resolution to infer the omics profiles (transcriptomes and proteomes) [60,63,64], while another study obtained hyperspectral Raman images of cells using galvo mirrors [62]. The optimal spatial resolution may depend on factors such as the size and structural complexity of the target cells, but clarifying the effect of spatial resolution on the accuracy of omics profile inference remains a future challenge. Similarly, there has been no systematic analysis of the contribution of spectral resolution to omics inference. High spectral resolution allows us to capture small peaks and fine structures of Raman spectra. However, since Raman-based inference of omics profiles exploits the statistical correlation between Raman spectral patterns and omics profiles and the effective low dimensionality of Raman and omics data, it remains elusive whether increasing spectral resolution will immediately lead to higher precision of inferred omics profiles. Characterizing the appropriate spectral resolution for omics inference would help to clarify the required optical configuration.

Lastly, the post-measurement data analysis methods also significantly affect the precision of omics inference. Therefore, the appropriate choice of analytical methods is crucial. The choice should depend on the number of samples and the noise level of the Raman spectra available for analysis. For example, nonlinear machine learning methods are generally effective with large datasets [62,87]. On the other hand, they are expected to be less effective and may perform poorly due to overfitting when applied to small datasets, although there is still active debate [124–127]. When available Raman spectral data is limited, simple linear methods may perform better than nonlinear methods. Noise in Raman spectra can also affect the accuracy of omics inference; appropriate prior denoising of Raman spectra before linking them to omics profiles can significantly improve accuracy. To facilitate future exploitation of Raman spectroscopic omics profiling, all these technical issues should be clarified and resolved.

So far, the arguments have implicitly assumed that omics profiles are more informative about cellular state differences than cellular Raman spectra. However, it might be interesting to ask which profile, Raman or a specific layer of omics, contains richer information. As they reflect the entire molecular composition of cells, it cannot be excluded that Raman spectra contain more unbiased and comprehensive information than specific omics layers such as transcriptome, proteome and metabolome (Fig. 5). Although many technical challenges remain to be overcome, future studies might utilize cellular Raman spectra as whole omics information along with other omics methods to interrogate diverse biological phenomena.
